# A Zika virus from America is more efficiently transmitted than an Asian virus by *Aedes aegypti* mosquitoes from Asia

**DOI:** 10.1038/s41598-017-01282-6

**Published:** 2017-04-27

**Authors:** Julien Pompon, Ronald Morales-Vargas, Menchie Manuel, Cheong Huat Tan, Thomas Vial, Jun Hao Tan, October M. Sessions, Pedro da Costa Vasconcelos, Lee Ching Ng, Dorothée Missé

**Affiliations:** 1 Programme in Emerging Infectious Diseases, Duke-NUS Medical School, Singapore, Singapore; 2 MIVEGEC UMR IRD 224-CNRS 5290-UM, IRD, Montpellier, France; 3 Department of Medical Entomology, Faculty of Tropical Medicine, Mahidol University, Bangkok, Thailand; 4 Environment Health Institute, National Environment Agency, Singapore, Singapore; 5 UMR 152 Pharma-DEV, IRD-Université de Toulouse, Toulouse, France; 6 000 0004 0620 4442 Department of Arbovirology and Hemorrhagic Fevers, Instituto Evandro Chagas, Ananindeua, Pará Brazil

## Abstract

Zika is a mosquito-borne disease associated with neurological disorders that causes an on-going pandemic. The first outbreak was recorded in Micronesia in 2007, then in French Polynesia in 2014 from which it spread to South America in 2015 and ignited a widespread epidemic. Interestingly, Zika outbreaks in Asia remained of moderate intensity although the virus is circulating. To understand these epidemiological variations, we investigated the entomological determinants of ZIKV transmission in Asia. We used oral infection of mosquitoes collected in Singapore to identify the vector species, to quantify the blood infection threshold and to compare transmissibility between an Asian ZIKV strain (H/PF13) and an American strain collected in Brazil (BE H 815744). We have confirmed the vector status of *Aedes aegypti* and determined that 10^3^ pfu/ml of blood is sufficient to infect mosquitoes. We showed that only the American strain was present in the saliva 3 days post-infection, and that this strain had a 30–40% higher rate of saliva infection in *Ae*. *aegypti* from 3 to 14 days post-infection than the Asian strain. Our data suggests that American strains are more efficiently transmitted than Asian strains, which raises concerns about the introduction of American strains in Asia.

## Introduction

ZIKA virus (ZIKV) is a newly emerging mosquito-borne virus that belongs to the Flavivirus genus of the *Flaviviridae* family. The virus can cause life-debilitating brain defects in adults and newborns from infected mothers^1–3^. ZIKV is responsible for an on-going pandemic that started in the Yap Island in 2007, then French Polynesia in 2014 and spread to South and Central America, where it resulted in a widespread epidemic^4–6^. Introduced on the American continent in early 2015, ZIKV has, in just two years, infected tens of thousands individuals and resulted in thousands confirmed cases of microcephaly in Brazil^7–9^. In response, the World Health Organization declared the cluster of microcephaly and other neurological disorder as a ‘Public Health Emergency of International Concern’ on the 1^st^ of February 2016^10^.

ZIKV is also present on the African and Asian continents but has not resulted in similarly large outbreaks, despite the presence of permissive mosquito vectors and favorable ecological conditions for transmission. Although the first human cases of ZIKA were detected in the 1950s in Uganda and Tanzania^11^, there have been no detectable outbreaks in Africa in the intervening years. For the past four decades, the virus has also been reported in many Southeast Asian countries^12, 13^. Since the emergence of the current epidemic in 2014, Singapore has reported its first autochthonous transmission in August 2016^14^. By the end of 2016, however, the ZIKV introduction had only resulted in a minor epidemic (457 reported cases) and no new cases were reported in the last three weeks of the year (www.nea.gov.sg). In Cambodia, a recent study reported that the virus was circulating with a low prevalence between 2007 and 2016^15^. While closely related genetically, Southeast Asian and American strains form different lineages^16^ and the Southeast Asian ZIKV might not be the direct source of the South American outbreaks^17^. A better characterization of the entomological determinants of ZIKV transmission in Asia could help understand the epidemiological variation between Asia and America.

ZIKV can be transmitted by *Aedes aegypti* and *Aedes albopictus* mosquito species^18–20^. ZIKV has also been reported to infect *Culex quinquefasciatus* ^21^, although this is highly controversial^22–24^ and may depend upon the origin of the mosquito colony and the virus strain. For instance, mosquito transmission efficiency for dengue virus, another epidemic flavivirus, depends upon mosquito origin^25, 26^ and mosquito-virus genetic interaction^27^.

Currently, there is no clear evidence to explain the relatively low prevalence of ZIKV infection in Southeast Asia, nor why there have been no large ZIKV outbreaks in Asia similar to those seen in South and Central America. Here, we endeavored to determine the entomological factors that might play a role in the epidemiology of ZIKV in Southeast Asia. Working with mosquito colonies collected in Singapore, we confirmed that *Ae*. *aegypti* mosquitoes are more susceptible to ZIKV infection than *Ae*. *albopictus* and ruled out *Cx*. *quinquefasciatus* as an efficient vector for ZIKV transmission. We also showed that *Ae*. *aegypti* is extremely susceptible to ZIKV infection by determining the blood infection threshold. Importantly, we found that a ZIKV strain from Brazil (BE H 815744) had higher transmissibility than a strain originating from the Asian lineage (H/PF13) in *Ae*. *aegypti*.

## Results

### *Aedes aegypti* is the main vector of Zika virus in Southeast Asia

**Figure 1:**
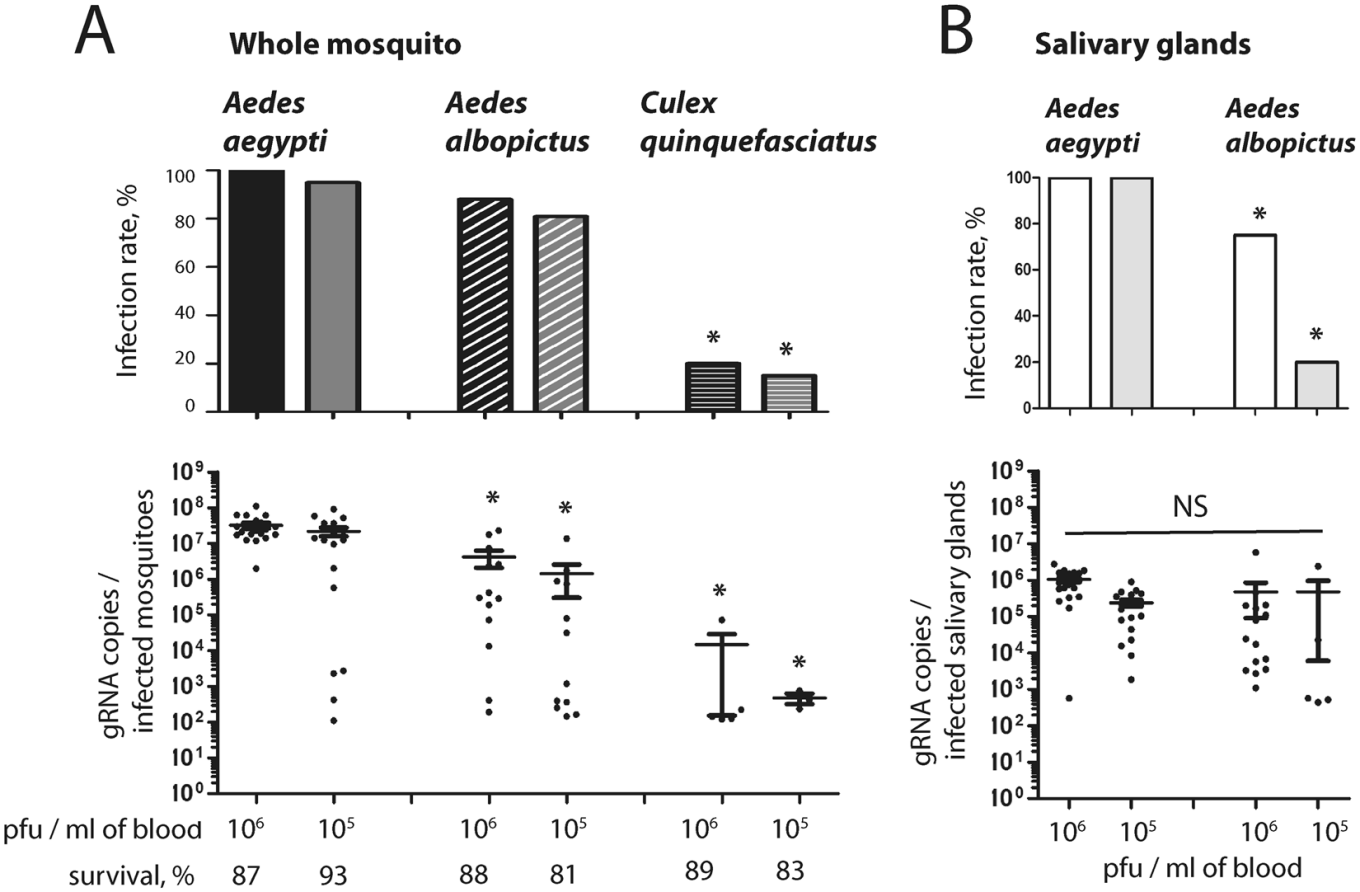
Comparison of susceptibility to Zika virus infection between *Ae*. *aegypti*, *Ae*. *albopictus* and *Cx*. *quinquefasciatus*. Mosquitoes were orally infected with PF13 at a titer of 10^6^ or 10^5^ pfu/ml. (**A**) At 7 days p.i., ZIKV genome copies were quantified in whole mosquitoes. (**B**) At 14 days p.i., ZIKV genome copies were quantified in salivary glands. Infection rate and gRNA copies per infected mosquitoes are shown. Each point represents one mosquito sample. Line shows average of gRNA copies. Twenty mosquitoes were tested per condition. Asterisks indicate significant difference with *Ae*. *aegypti* in the same condition following a Z-test and T-test for infection rate and gRNA copies, respectively.

To identify the main vector of ZIKV in Southeast Asia, we compared the susceptibility of *Ae*. *aegypti*, *Ae*. *albopictus* and *Cx*. *quinquefasciatus* mosquitoes from Singapore to the French Polynesian (H/PF13) ZIKV strain that is representative of the Asian lineage^13^. The mosquitoes were orally fed with a mix of blood containing 10^6^ or 10^5^ pfu/ml in order to determine the influence of the inoculum concentration. At 7 days post-infection (p.i.), we detected and quantified the virus genome copies in whole mosquitoes. Infection rate and genome copies in *Cx*. *quinquefasciatus* were significantly lower than in *Ae*. *aegypti* for the two virus titers tested (Fig. 1A). Infection rates in *Ae*. *albopictus* mosquitoes were not different from *Ae*. *aegypti*, however the number of genome copies was significantly lower in *Ae*. *albopictus*.

Transmission of arboviruses is dependent upon infection of salivary glands^28^. To differentiate the vector potential of *Ae*. *aegypti* and *Ae*. *albopictus*, we orally infected the two species with 10^6^ or 10^5^ pfu/ml and quantified the virus genome copies at 14 days p.i. in dissected salivary glands. While genome copies were not different between the two species, infection rate was significantly higher in salivary glands of *Ae*. *aegypti* (Fig. 1B). These results clearly demonstrate that *Ae*. *aegypti* is the main vector of ZIKV in Singapore and probably in Southeast Asia.

### A very low blood titer threshold is required to infect *Ae*. *aegypti* mosquitoes

**Figure 2:**
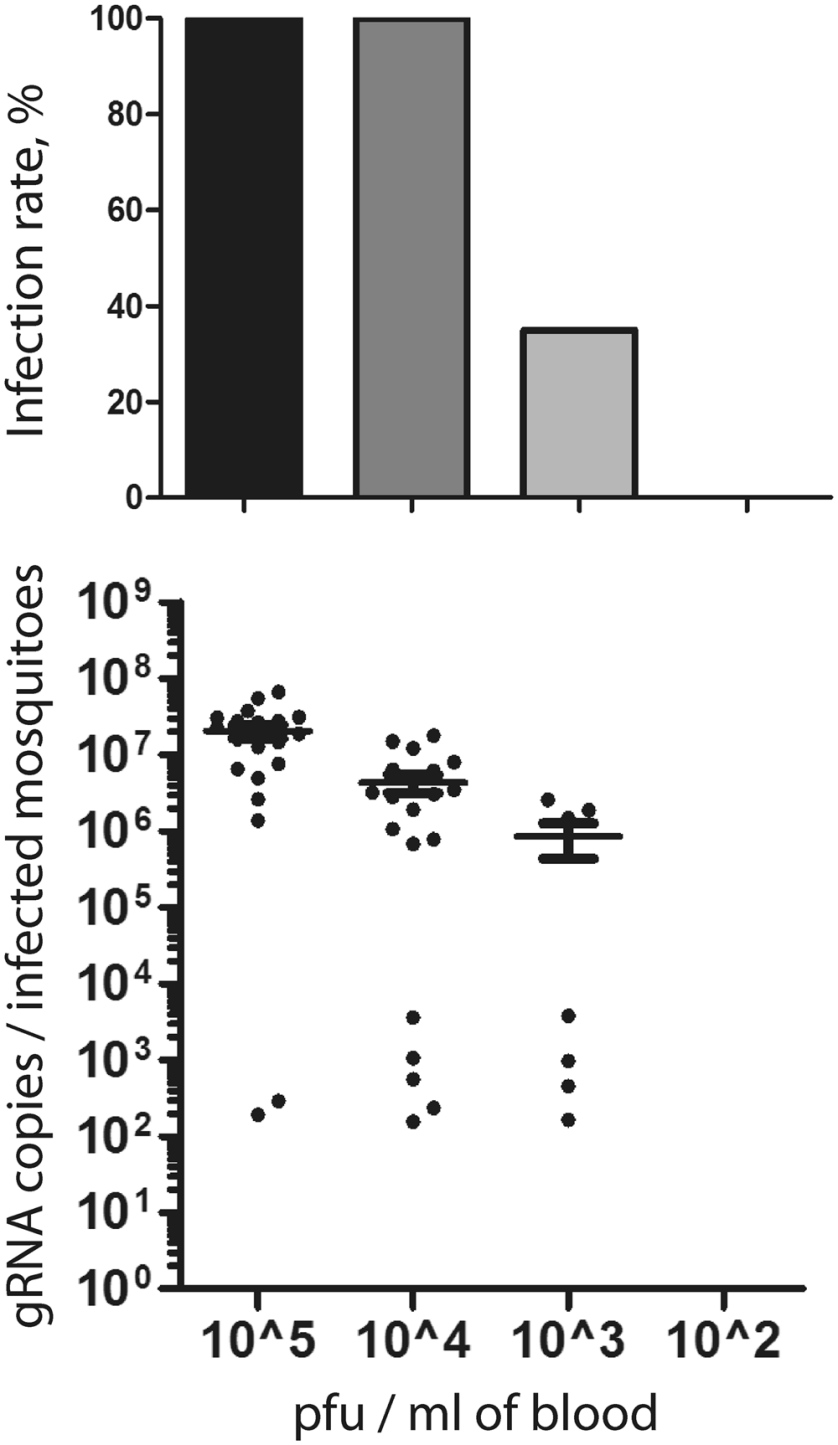
Zika virus can infect mosquitoes at a titer of 10^3^ pfu/ml in the blood. *Aedes aegypti* mosquitoes were orally infected with 10^5^, 10^4^, 10^3^ or 10^2^ pfu of PF13 per ml of blood. At 7 days p.i., ZIKV genome copies were quantified in whole mosquitoes. Each point represents one mosquito sample. Twenty mosquitoes were tested per condition. Line shows average of gRNA copies.

The capacity of the virus to infect mosquitoes greatly influences its transmission efficiency^28^. To determine the titer threshold for mosquito infection, we orally infected *Ae*. *aegypti* with H/PF13 at 10^5^, 10^4^, 10^3^ or 10^2^ pfu/ml of blood and quantified the virus genome copies in whole mosquitoes at 7 days p.i. All mosquitoes were infected up to 10^4^ pfu/ml, 35% were infected with a titer of 10^3^ pfu/ml and no mosquitoes were infected after feeding on 10^2^ pfu/ml of blood (Fig. 2). ZIKV genome copies per infected mosquito gradually decreased in a dose-dependent manner. Average virus genome copies varied from 2 × 10^7^, 4 × 10^6^ to 8 × 10^5^ after infection with 10^5^, 10^4^ and 10^3^ pfu/ml, respectively. Our data determined the threshold of infection in *Ae*. *aegypti*.

### A strain from Brazil is more efficiently transmitted by *Ae*. *aegypti* than a strain from French Polynesia

**Figure 3:**
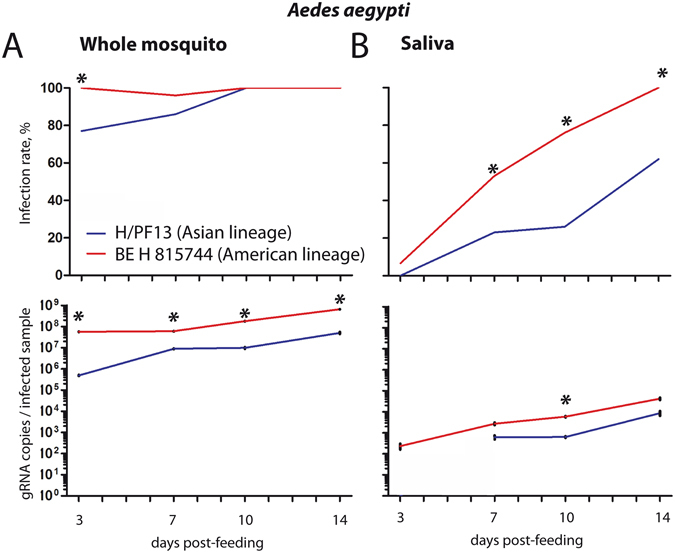
*Aedes aegypti* mosquitoes infected with a strain from Brazil have a higher infection rate of saliva. *Aedes aegypti* mosquitoes were orally infected with 10^5^ pfu/ml of PF13 or the Brazil virus. At 3, 7, 10 and 14 days p.i., Zika virus genome copies was quantified in (**A**) whole mosquitoes and (**B**) saliva. Percent of infection rate and average ± s.e.m. for genome copies per infected mosquitoes or saliva are presented. Thirty mosquitoes were analyzed per condition. Asterisks show differences between the two viruses within the same day following a Z-Test and T-test for infection rate and gRNA copies, respectively.

The extrinsic incubation period corresponds to the time between oral infection and presence of virus in the saliva of vectors. This period determines transmission efficiency of arboviruses^28^. To compare the efficiency of transmission for viruses from the Asian and the American lineages, we orally infected *Ae*. *aegypti* mosquitoes with H/PF13 (Asian lineage) or BE H 815744 (American lineage) with 10^5^ pfu/ml of blood. At 3, 7, 10 and 14 days p.i., we quantified virus genome copies in whole mosquitoes and in saliva. In whole mosquitoes, BE H 815744 showed a higher infection rate early during our study period (day 3 p.i.), and had higher genome copies at each time point (Fig. 3A). To confirm this result, we titered the virus in whole mosquitoes at 3 days p.i. with H/PF13 or BE H 815744. Similarly to our results obtained with virus genome quantification, virus titer was significantly higher in mosquitoes infected with BE H 815744, although titers for both viruses were several orders lower than genome copies (Fig. S1). Strikingly, in the saliva, the Brazilian strain was present earlier (day 3 p.i.) and had higher rate of infection than H/PF13 at the other time points (Fig. 3B). Although only significant at 10 days p.i., the American strain had consistently higher average number of genome copies per infected saliva. These results indicate that the American strain BE H 815744 could be more efficiently transmitted than the Asia lineage strain H/PF13.

## Discussion

The on-going ZIKA pandemic continues to spread throughout the tropics and sub-tropics but has not caused outbreaks of similar magnitude in all regions. As with other pathogenic Flaviviruses, ZIKV is transmitted through the bite of a mosquito that inoculates the virus along with its saliva when biting. Additionally, ZIKV can be transmitted through sexual intercourse^9, 29, 30^ and between the mother and her foetus^31^. While the rapid expansion of the virus across the tropical world is indicative of efficient transmission, variation in transmissibility could account for the epidemiological variation^9^. ZIKV is currently detected in Southeast Asia but has not been associated with outbreaks of the same scale as those observed in South and Central America. Our data reveal that, despite the presence of a very efficient vector in Southeast Asia, the Asian ZIKV strains may not be as efficiently transmitted as the American strains.

The vector status of mosquito species depends on their competence to transmit the virus^28^. Vector competence is determined by the ability of the virus to replicate to high titers throughout the mosquito body and particularly in the salivary glands, thus facilitating transmission during subsequent blood feeding^32^. Comparing *Ae*. *aegypti*, *Ae*. *albopictus* and *Cx*. *quinquefasciatus* mosquitoes collected in Singapore, we found that salivary glands of *Ae*. *aegypti* had the highest infection rate across different ZIKV blood titers. Furthermore, *Ae*. *aegypti* mosquitoes were susceptible to infection by ZIKV down to a titer of 10^3^ pfu/ml of blood at 7 days p.i. Previous estimates have quantified that *Ae*. *aegypti* blood meal size is on average 2.8 µl^33^, suggesting that as little as 2.8 infectious particles are enough to infect a mosquito. For comparison, the infection threshold for twelve strains of dengue virus was determined in *Ae*. *aegypti* collected in Thailand^34^; infection rate was not higher than 15% 7 days p.i. after feeding on a blood titer of 10^3.7^ pfu/ml. Our data confirmed the vector status of *Ae*. *aegypti* and provide new insight about the ZIKV infectivity to mosquitoes.

Though significantly lower than for *Ae*. *Aegypti*, our results have also shown a high rate of salivary gland infection for *Ae*. *albopictus* originating from Southeast Asia. *Aedes albopictus* mosquitoes have been spreading through Brazil for decades^35^ and its potential contribution to the outbreaks should also be taken into account. ZIKV has been detected in field-caught *Ae*. *albopictus* from Gabon^36^. Additionally, a number of other *Aedes* species mosquitoes are susceptible to ZIKV; viral isolations were reported from many species of *Aedes* in Africa^37–39^. For *Cx*. *quinquefasciatus*, our data clearly indicate that it is not a competent vector in Singapore.

Flaviviruses have an error-prone replication system that can result in rapid evolution of their genome and generate new strains with variable epidemiological fitness. For instance, variation in transmission efficiency between dengue viruses can result in different epidemiological fitness^34, 40^. Here, we showed that a strain from Brazil (BE H 815744) had a higher transmissibility than a strain from the Asian lineage (H/PF13); the Brazilian strain had a shorter extrinsic incubation period and higher infection rate of saliva. To identify nucleotide variations that could account for the differences in vector competence, we compared H/PF13 and BE H 815744 genome sequences (Table 1). We first sequenced BE H 815744 from our virus stock to control for mutations acquired during expansion and although we did detect thirty mutations relative to the reference sequence (KU365780), all but two of these were conservative and did not affect protein coding (Table S1). Additionally, we also examined the low-frequency variants present in the viral inoculum and our results indicated that no further positions in the genome had greater than 6% divergence from the published sequence (Table S2)^41^. Overall, there were 5 nucleotides that differed between H/PF13 and BE H 815744 and changed the amino acid sequence, and 2 of these were fixed in Asian and American strains for which the whole genome was available (Table 1; Table S3). These two segregating substitutions were located in NS4B and 3′UTR. Phylogenetic analysis previously identified an on-going selection pressure on the NS4B gene^42^, which inhibits the innate immune response^43^. The 3′UTR of all flaviviruses produces a subgenomic flaviviral RNA (sfRNA) upon partial degradation of their genomic RNA^44, 45^. SfRNA can alter the innate immune response in both mammals and mosquitoes^44, 46^ and single mutations in 3′UTR sequence of dengue virus have been linked to increased epidemiological fitness by altering the interferon innate immune response in human^47^. While additional American and Asian strains need to be tested, we suggest that epidemiological differences between the Americas and Asia are due to variation in mosquito transmission efficiency and we highlighted fixed nucleotide differences between lineages. The ZIKV strains that caused the recent moderate outbreaks in Singapore^48^, Thailand^49^ and Cambodia^15^ all belonged to the Asian lineage. Our study then raises concerns about the introduction of American ZIKV strains in Asia where there are competent mosquito vectors and climatic conditions suitable for transmission.

**Table 1:** Non-synonymous substitutions between PF13 and BE H 815744, and the distribution of these substitutions in strains from America and Asia.

Gene	Position^b^	Nucleotide substitution (aa substitution)		Nucleotide substitution; Presence/number of observed strains^a^	
		H/PF13	BE H 815744	Asian strains	American strains
PrM	859	C (L)	T (F)	T; 7/7	T; 34/34
NS1	2908	G (E)	A (K)	A; 7/7	G; 1/34 - A; 33/34
	3517	A (M)	G (V)	A; 7/7	A; 29/34 - G; 5/34
NS4B	7990	A (M)	G (V)	A; 7/7	G; 34/34
3′UTR	10374	G	A	G; 7/7	A; 34/34

^a^We selected strains for which the complete genome was available in NCBI and that were collected in epidemic countries (to avoid imported cases; strains collected in China and Japan were not included) in America and Asia after 2010. See Table S3 for detail. ^b^Position was defined according to H/PF13.

## Material and Methods

### Mosquito colonies

The *Ae*. *aegypti*, and *Ae*. *albopictus* colonies were established in 2010 from eggs collected in Singapore. *Culex quinquefasciatus* colony were derived from larvae collected by enforcement officers of the National Environment Agency (NEA), Singapore from residential premises during routine house to house inspections in 2010. Eggs were hatched in tap water, larvae were fed a mix of fish food (TetraMin fish flakes) and liver powder (MP Biomedicals), and adults were held in rearing cages (Bioquip) supplemented with 10% sucrose and fed pig’s blood (Primary Industries Pte Ltd) twice weekly. The insectary was held at 28 °C with 50% humidity on a 12:12 h dark:light cycle.

### Virus isolates

H/PF13 (H/PF/2013) was collected from human serum in French Polynesia in 2013 and obtained from the European Virus Archive (EVA). The Brazilian strain (BE H 815744) was collected in the Paraiba state (northeast region), Brazil, in 2015 from a febrile non-pregnant woman with a rash. Viruses were propagated in C6/36 cells and used after 6 and 3 passages for H/PF/13 and BE H 815744, respectively. Viruses were titrated trice by plaque assay in BHK-21 cells as previously described^50^.

### Oral infection

Three to five day-old mosquito females were sugar-deprived for 24 h and subsequently offered a blood meal containing a 40% volume of washed erythrocytes from SPF pig’s blood (PWG Genetics), 5% of 100 mM ATP (Thermo Scientific), 5% human serum (Sigma) and 50% volume of virus in RPMI (Gibco). The blood viral titers for both ZIKV strains were confirmed by plaque assays^50^. Mosquitoes were exposed to the artificial blood meal for one hour using a Hemotek membrane feeder system (Discovery Workshops) with a porcine intestine membrane. Fully engorged females were selected and maintained with free access *ad libitum* to a 10% sugar solution in an incubation chamber with conditions similar for insect rearing until analysis.

### Saliva collection

Orally infected mosquitoes were cold-anesthetized and severed from their wings and legs. Their proboscis was inserted into a 10 µl filter tip containing 10 µl of SPF blood. Mosquitoes were allowed to expectorate for 30 min.

### Real-Time quantitative PCR quantification of Zika virus genome copies

Single mosquitoes or total volume of blood used to collect saliva were homogenized in 350 µl of TRK lysis buffer (E.Z.N.A. Total RNA kit I (OMEGA Bio-Tek)) using a bead Mill homogenizer (FastPrep-24, MP Biomedicals) for mosquitoes and by pipetting for blood. Total RNA was extracted using E.Z.N.A. Total RNA kit I (OMEGA Bio-Tek) and eluted in 30 µl of DEPC-treated water. Genomic RNA (gRNA) copies was quantified with a one-step RT-qPCR with Sensifast SYBR No-ROX one-step kit (BioLine). Primers, targeting conserved region in the envelope, were: 5′- AGGACAGGCCTTGACTTTTC-3′ and 5′-TGTTCCAGTGTGGAGTTC-3′. The 10 µl reaction mix contained 400 nM of forward and reverse primers, and 3 µl of RNA extract. Quantification was conducted on a CFX96 Touch Real-Time PCR Detection System (Bio-Rad). Thermal profile was 45 °C for 10 min, 95 °C for 1 min and 40 cycles of 95 °C for 5 sec and 60 °C for 20 sec.

An absolute standard curve was generated by amplifying fragments containing the qPCR targets using the qPCR forward primers tagged with a T7 promoter and the qPCR reverse primer. The fragment was reverse transcribed using MegaScript T7 transcription kit (Ambion) and purified using E.Z.N.A. Total RNA kit I. The total amount of RNA was quantified using a Nanodrop (ThermoScientific) to estimate copy number. Ten times serial dilutions were made and used to generate an absolute standard equation. In each subsequent RT-qPCR plate with samples, we quantified four standard aliquot dilutions to adjust for threshold variation between plates.

### Virus Titration

Individual mosquitoes were homogenized in 500 µl of RPMI, filtered through 0.22 µm filter (Sartorius) and titered using plaque assay with BHK-21(ATCC® CCL-10) cells as previously described^47^.

### Virus Sequencing

Total RNA from 500 µl of the virus stocks were extracted using RNAzol RT (MRC). Library preparation was done using NEBNext Ultra Directional RNA Library Prep Kit (NEB) and sequenced on an Illumina MiSeq instrument at the Duke-NUS Genome Biology Facility. Quality control was performed on the paired-end raw reads using FastQC v0.10.1^51^ and trimmed using Trim Galore v0.4.0. These reads were then competitively mapped against all publicly available complete ZIKV genomes from NCBI using BWA v0.7.12-r1039^52^ to identify the best initial reference with the highest number of mapped reads, using SAMtools v0.1.19^53, 54^ “idxstats” command. The consensus sequence was then generated using “bam2cons.py v0.1” script from the Viral Pipeline Runner (ViPR), with parameter setting “MIN_COV = 0” and the best initial reference.

### Virus genome from NCBI

Zika Virus Resource from NCBI was searched for complete genome sequences for Zika virus from Asia and America on 22^nd^ December 2016 (Table S1). We excluded sequences from virus collected in Japan and China as they were potentially imported cases. H/PF13 (H/PF/2013) and BE H 815744 codes were KX369547 and KU365780, respectively.

## Electronic supplementary material

Supplementary material
