# Supplementary material for: A Zika virus from America is more efficiently transmitted than an Asian virus by Aedes aegypti mosquitoes from Asia
Source: Sci Rep. 2017 Apr 27;7:1215. doi: 10.1038/s41598-017-01282-6 (PMC5430906; doi:10.1038/s41598-017-01282-6)
Supplement: Supplementary file 1 — Supplementary material [file 41598_2017_1282_MOESM1_ESM.pdf]

SUPPLEMENTARY MATERIAL

A Zika virus from America is more efficiently transmitted than an Asian virus by *Aedes aegypti* mosquitoes from Asia

Short title: Difference in ZIKV transmissibility between American and Asian strains

Julien Pompon[1,2,*], Ronald Morales-Vargas[3], Menchie Manuel[1], Cheong Huat Tan[4], Thomas Vial[5], Jun Hao Tan[1], October M. Sessions[1], Pedro da Costa Vasconcelos[6], Lee Ching Ng[4], Dorothée Missé[2,*]

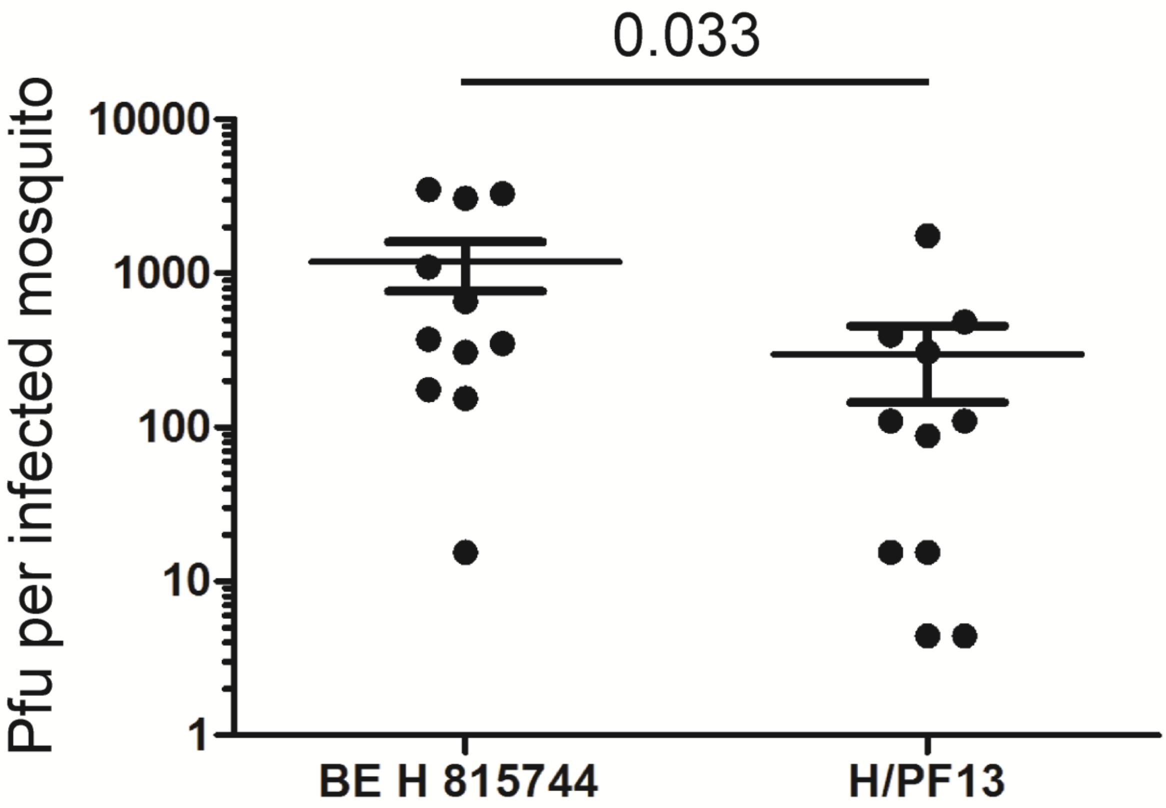

Figure S1. Virus titer in whole mosquitoes 3 days post-infection with ZIKV from the American (BE H 815744) or Asian (H/PF13) lineage.

Mosquitoes were orally infected with the two viruses and collected for titration 3 days p.i. Each point represents a mosquito. T-test statistical difference is shown.

Table S1. Summary of consensus level changes between the virus inoculum and the published KU365780 sequence

Position	Reference (KU365780)	Virus inoculum	Amino Acid coding	Codon changed in the virus stock ?
836	C	T	H	no
863	U	C	F	no
923	G	A	T	no
1034	C	T	T	no
1433	U	C	I	no
1475	A	G	K	no
1904	U	C	T	no
3137	U	C	H	no
**3534**	**A**	**G**	**M --> V**	**yes**
3566	C	T	D	no
5267	C	T	R	no
5270	C	T	T	no
5315	G	A	E	no
5438	A	G	R	no
6320	C	T	N	no
6671	C	T	F	no
6803	U	C	F	no
6959	U	C	H	no
6983	G	A	G	no
7493	G	A	G	no
8213	G	A	K	no
8327	U	C	Y	no
8408	U	A	P	no
**8439**	**G**	**A**	**D --> N**	**yes**
8867	C	T	F	no
8978	U	C	D	no
9242	U	C	R	no
9344	A	G	E	no
9560	A	G	L	no
10447	-	C	N/A	N/A
836	C	T	H	no
863	U	C	F	no
923	G	A	T	no
1034	C	T	T	no
1433	U	C	I	no
1475	A	G	K	no
1904	U	C	T	no
3137	U	C	H	no

**3534**	**A**	**G**	**M --> V**	**yes**
3566	C	T	D	no
5267	C	T	R	no
5270	C	T	T	no
5315	G	A	E	no
5438	A	G	R	no
6320	C	T	N	no
6671	C	T	F	no
6803	U	C	F	no
6959	U	C	H	no
6983	G	A	G	no
7493	G	A	G	no
8213	G	A	K	no
8327	U	C	Y	no
8408	U	A	P	no
**8439**	**G**	**A**	**D --> N**	**yes**
8867	C	T	F	no
8978	U	C	D	no
9242	U	C	R	no
9344	A	G	E	no
9560	A	G	L	no
10447	-	C	N/A	N/A

Table S2. Analysis of low frequency variants in the viral inoculum

Position	Consensus	Variant	Depth of Coverage	Allele frequency
326	G	A	14572	0.0122
436	T	C	20630	0.0051
1028	T	G	23218	0.0067
1278	T	C	21663	0.0064
2400	T	C	18196	0.0058
3147	A	G	22397	0.0054
3535	T	C	22939	0.0581
4106	A	G	19790	0.0055
7416	A	G	16086	0.0052
7499	T	C	11179	0.0062
9227	A	G	22884	0.0071
9434	A	G	20014	0.0065
9548	T	C	20185	0.0051
9917	C	A	16861	0.0199
10220	T	C	17801	0.0050
10666	T	A	11990	0.0067
10717	A	G	7013	0.0093
10772	A	G	2834	0.0159

Table S3. Code and origin of the strain used for genome comparison

Continent	Country	Date of collection	GenBank ID
Americas	Brazil	2015	KU365780[1]
			KX280026
			KU527068
			KU321639
			KU497555
			KX197205
			KU365778
			KX520666
			KR872956
			KU365780
			KU365777
			KU365779
			KU707826
			KU729217
			KU729218
		2016	KY014320
			KY014307
			KY014317
			KY014301
			KY014297
			KX197192
			KX811222
			KY014296
	Columbia	2015	KU820897
			KX087102
			KX548902
		2016	KX247646
	Ecuador	2016	KX879603
			KX879604
	French Guiana	2015	KU758877
	Suriname	2015	KU312312
		2016	KU937936
	Venezuela	2016	KX893855
			KX702400
Asia	Cambodia	2010	KU955593
	French Polynesia	2013	KX369547[1]
	Philippines	2012	KU681082
	Thailand	2013	KX694532
		2014	KU681081
		2016	KY272987
	Singapore	2016	KX827309
			KX813683

[1] Virus used in the oral infection study.
